# Regulatory Roles of Bone in Neurodegenerative Diseases

**DOI:** 10.3389/fnagi.2020.610581

**Published:** 2020-12-21

**Authors:** Zhengran Yu, Zemin Ling, Lin Lu, Jin Zhao, Xiang Chen, Pingyi Xu, Xuenong Zou

**Affiliations:** ^1^Guangdong Provincial Key Laboratory of Orthopaedics and Traumatology, Orthopaedic Research Institute/Department of Spine Surgery, The First Affiliated Hospital of Sun Yat-sen University, Guangzhou, China; ^2^Department of Neurology, The First Affiliated Hospital of Guangzhou Medical University, Guangzhou, China

**Keywords:** bone, bone marrow, neurodegenerative diseases, mesenchymal stem cells, immune, osteokines

## Abstract

Osteoporosis and neurodegenerative diseases are two kinds of common disorders of the elderly, which often co-occur. Previous studies have shown the skeletal and central nervous systems are closely related to pathophysiology. As the main structural scaffold of the body, the bone is also a reservoir for stem cells, a primary lymphoid organ, and an important endocrine organ. It can interact with the brain through various bone-derived cells, mostly the mesenchymal and hematopoietic stem cells (HSCs). The bone marrow is also a place for generating immune cells, which could greatly influence brain functions. Finally, the proteins secreted by bones (osteokines) also play important roles in the growth and function of the brain. This article reviews the latest research studying the impact of bone-derived cells, bone-controlled immune system, and bone-secreted proteins on the brain, and evaluates how these factors are implicated in the progress of neurodegenerative diseases and their potential use in the diagnosis and treatment of these diseases.

## Introduction

Neurodegenerative diseases are characterized by the gradual loss of structure and function of selectively vulnerable neurons in different regions of the brain, affecting millions of people worldwide. Neurodegenerative disorders can be clinically identified by their representative features, mostly exhibiting extrapyramidal and/or pyramidal movement disorders, cognitive or behavioral disorders. Most patients suffer multiple clinical symptoms rather than pure phenotype (Dugger and Dickson, 2017). The related mechanism of neurodegenerative diseases has been studied. For example, the most common kind of dementia worldwide, Alzheimer’s disease (AD), is pathologically characterized by the extracellular amyloid-β (Aβ) peptides accumulation in senile plaques and the formation of intracytoplasmic neurofibrillary tangles in the brain (Querfurth and LaFerla, 2010; Livingston et al., 2017). And Parkinson’s disease (PD) is characterized by loss of dopaminergic neurons and insufficient synthesis of dopamine in the substantia nigra (SN) area (Dauer and Przedborski, 2003; Maiti et al., 2017), and the accumulation of intracytoplasmic α-synuclein polymers (Lewy bodies) in the brain (Goldman et al., 1983). Multiple sclerosis (MS) which shows both neuroinflammatory and neurodegenerative characteristics had been found it results from chronic demyelinating of the central nervous system (CNS; Compston and Coles, 2008). For the majority of MS patients (85–90%), tissue damage is more caused by inflammation rather than neuronal degeneration in the first 5–12 years of onset, whereas the balance between inflammatory response and neuronal degeneration shifts to the latter at the secondary stage of progression. However, for a minor proportion of MS patients (10–15%), neuronal degeneration plays a major role throughout the disease (Steinman, 2009).

With the increasing trend of social aging, neurodegenerative diseases and osteoporosis have become severe social issues. The bone-related effect in neurodegenerative diseases has so far gained an increased awareness of several epidemiological findings. A meta-analytic study showed that compared with healthy controls, AD patients have a lower hip bone mineral density and are more likely to suffer from hip fractures (Zhao Y. et al., 2012). Also, osteoporosis is regarded as a risk factor for AD, since osteoporosis correlates with the cognitive deficit and its severity in adults older than 50 (Zhou et al., 2011; Kang et al., 2018). Many PD patients have compulsory trunk scoliosis, known as Pisa syndrome, which causes severe late complications of disability (Poewe et al., 2017). In a more common scenario, AD or PD patients suffer from bone fracture, osteoarthritis, restricted movement, bed ulcer, and even death caused by severe osteoporosis. Osteoporosis and an abnormal amount of bone-derived factors in turn promote the progression of AD and PD, especially in women over 60 years old (Yuan et al., 2019).

Bone is a multifaceted, dynamic tissue, which participated in movement facilitation, mineral metabolism, immune cell generation, and mesenchymal stem cells (MSCs) or hematopoietic stem cells (HSCs) breading. Since the bone marrow (BM) contains enormous amounts of MSCs and serves as the primary lymphoid organ, both of which have already been proved to have effects on the brain by many studies, the significance of bone in the regulation of CNS homeostasis is un-doubtable. Moreover, some hormones or “osteokines” from bone cells such as osteocalcin (OCN), osteopontin (OPN), and fibroblast growth factor (FGF) 23 have endocrine functions (Vervloet et al., 2014; Han Y. et al., 2018). These proteins can act on the brain directly or indirectly by regulating phosphate homeostasis or systemic energy metabolism (Han Y. et al., 2018; Yuan et al., 2019). This review aims to clarify the inter-relationship between the bone and brain and to discuss its potential therapeutic implications for neurodegenerative diseases.

## BM-Derived Cells

**Figure 1:**
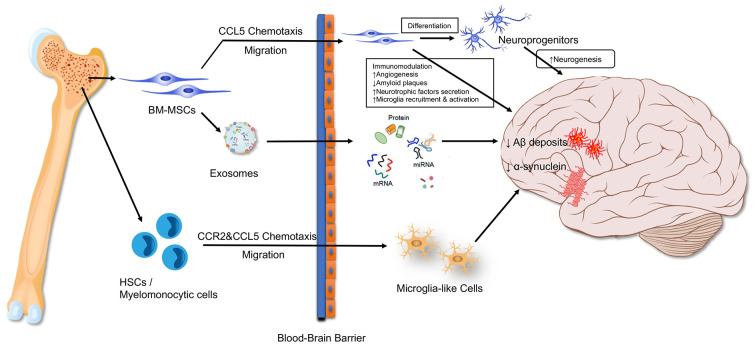
Bone marrow-derived cells influence the brain. BM-MSC, bone marrow mesenchymal stem cell; HSC, hematopoietic stem cell.

The bone organ consists of bone and Bone marrow(BM), both of which function as a single unit (Compston, 2002). BM is a soft and viscous tissue enclosed within the bone cortex, containing HSCs and MSCs. HSCs are responsible for producing blood cells, including leukocytes, monocytes, erythrocytes, and platelets, while MSCs are contributed to osteoclasts and osteoblasts’ origination. These stem cells are tightly involved in the neurodegenerative progressions. Transplantation of young BM can rejuvenate the hematopoietic system and preserve cognitive function in old recipient mice, suggesting the beneficial effects of BM to the brain (Castellano et al., 2015; Das et al., 2019). Several pathways are contributed to the beneficial effects such as these BM-derived cells migrating directly into the brain to exert their toxicant clearance, immuno-modulative and neuro-generative functions, or *via* secreting exosomes to enter the brain and execute their biological functions (Soulet and Rivest, 2008; Dennie et al., 2016; Nakano et al., 2016; Han K. H. et al., 2018; Figure 1).

### BM-Derived Microglia-Like Cells

Microglia are the primary immune effector cells in the CNS. These cells are monocytes that originated from mesodermal that invade the developing CNS in the embryonic period when the blood-brain barrier (BBB) is not fully formed. In addition to the primary resident microglia, another type of microglia that originates from precursors cells in the BM has been identified in the brain (Soulet and Rivest, 2008). To distinguish them from the originally brain-resident microglia cells, microglial cells originating from the outside of the brain are referred to as “microglia-like cells” (Han K. H. et al., 2018). Although BM-derived microglia-like cells can enter the CNS under normal physiological conditions, they are preferentially presented in regions suffering from neurodegeneration or exogenous insults (Soulet and Rivest, 2008). In the case of ischemic stroke, peripheral monocytes can migrate into the brain at the injury site and differentiate into ramified microglial-like cells (Priller et al., 2001a,b; Soulet and Rivest, 2008). This is even true for facial nerve axotomy and hypoglossal nerve axotomy models, in both cases the mouse models’ BBBs are not damaged (Priller et al., 2001a,b; Soulet and Rivest, 2008). *In vitro* studies showed Aβ-eliminating microglia-like cells could be differentiated from the BM-derived HSCs (Kuroda et al., 2020b) monocyte (Simard et al., 2006) and CD11b- or CD115-positive cells (Lebson et al., 2010; Koronyo et al., 2015). Further mechanism study showed that cells from the human and mouse BM can become microglia-like cells under the stimulation of colony-stimulating factor-1 (Kuroda et al., 2020a). An *in vivo* study showed that BM-derived microglia-like cells can move through the BBB and accumulate inside the brain in a CCR2 chemokine-dependent manner (El Khoury and Luster, 2008). The decreased CCR2 expression can reduce microglia accumulation and increase the level of Aβ in the brain, suggesting that early microglial accumulation can promote Aβ clearance in AD mouse models (El Khoury and Luster, 2008).

We reviewed the BM-derived microglia-like cells’ contribution to microgliosis in neurodegenerative diseases. Researchers transferred GFP-labeled myeloid cells into the BM of irradiated AD mice, and these GFP-labeled cells were later detected in the senile plaques in the brains (Simard et al., 2006). Similarly, early and rapid recruitment of BM-derived microglia to the brain also occurs in prion disease, bacterial meningitis, and PD mouse models (Kokovay and Cunningham, 2005; Djukic et al., 2006). Intracerebral injection of externally differentiated BM-derived microglia-like cells can also decrease Aβ deposits and improved cognitive function in AD mouse models (Kawanishi et al., 2018). These injected microglia-like cells can automatically migrate toward Aβ plaques, and reduce the number and area of these plaques (Lampron et al., 2011). Another study used a novel transgenic AD mouse to demonstrate that it is the peripheral blood-originated microglia, rather than their resident counterparts, that are capable of phagocytosing Aβ deposits (Simard et al., 2006). Besides, BM-derived microglia-like cells could also stimulate the phagocytic functions of brain-resident microglia *in vitro* and *in vivo*, *via* secreting large sums of transforming growth factor-β (TGF-β; Kuroda et al., 2020a). All these results suggest that BM-derived microglia-like cells have potential cell-based disease-modifying therapy against neurodegenerative diseases, especially AD.

### BM-MSCs

Bone marrow MSCs (BM-MSCs) are pluripotent stem cells inside the BM. It has been proved that BM-MSCs can move into the brain and develop neuronal markers such as nestin, doublecortin, and NeuN under physiological conditions (Dennie et al., 2016). The CCR5 plays a critical role in regulating the BM-MSCs’ migration into the brain, both in physiological conditions and in response to injury (Dennie et al., 2016). Thus, BM-derived progenitors can migrate to the brain and become neurons at least in part, by firstly differentiating into neuronal precursor cells (Dennie et al., 2016). Apart from that, BM-MSCs can migrate to the brain and exert strong neuroprotective effects *via* regulating neurogenesis, apoptosis, angiogenesis, immunomodulation, and eliminating Aβ plaques in the brain (Naaldijk et al., 2017; Qin et al., 2020).

Transplanted BM-MSCs could significantly diminish the hippocampal Aβ plaques by activating several Aβ-degrading enzymes (Jha et al., 2015). Since vascular damage is also a pathogenic factor of AD, BM-MSCs can promote angiogenesis in the AD brain by secreting vascular endothelial growth factor (VEGF), epidermal growth factor (EGF), FGF-2, and Ang-1 (Gallina et al., 2015), and thus favor the cognitive and behavioral recovery (Garcia et al., 2014). The MSCs can also exert neuroprotective effects by secreting neurotrophic factors such as neurotrophin-3 (NT-3), hepatocyte growth factor (HGF), and brain-derived neurotrophic factor (BDNF; Wang et al., 2010). These factors stimulate endogenous regeneration and contribute to neurobehavioral function recovery. Animal studies demonstrate that the immunomodulatory effect of BM-MSCs plays a vital role in AD treatment as well (Salem et al., 2014; Zhang et al., 2020). Transplanted BM-MSCs can attract microglia-like cells by secreting CCL5 both *in vitro* and in the AD brain (Lee J. K. et al., 2012). Despite this, the activation levels of microglia and astrocyte were decreased in AD mice brains after BM-MSCs transplantation, manifested as the cerebral Iba-1 levels were down-regulated (Yokokawa et al., 2019). Our previous study showed that the transplanted BM-MSCs could inactivate microglia in the peri-infarct area *via* the CD200-CD200R1 signaling (Li et al., 2019). Also, BM-MSCs could lower expressional levels of pro-inflammatory genes, cytokines, and enzymes in astrocytes (Schäfer et al., 2012; Naaldijk et al., 2017). However, other studies report that the BM-MSCs could accelerate the microglia activation and thus the Aβ clearance in the AD brain (Lee et al., 2009). The BM-MSCs could also significantly increase the number of ChAT-positive cells and the intensity of ChAT spots in AD brains, which is an indicator for neurogenesis, neuronal differentiation, and integration (Mezey and Chandross, 2000; Mezey et al., 2000; Sanchez-Ramos et al., 2000). Nestin in neural cells, a neural precursor biomarker, is also up-regulated in the brain following the BM-MSC transplantation (Sanchez-Ramos et al., 2000). In fact, cells in different neurogenic stages, including proliferation, differentiation, migration, targeting, and integration, could be found in the hippocampus after the BM-MSC treatment (Perry et al., 2012).

Furthermore, BM-MSCs can alleviate cognitive defects related to various neurological disorders including traumatic brain injury (TBI), PD, and stroke by secreting microvesicles (MV) or exosomes (Xiong et al., 2017; Yang et al., 2017). It has been hypothesized that vesicles from BM-MSCs facilitate the transference of various functional factors, including regulatory non-coding RNAs, lipids, and proteins (Reza-Zaldivar et al., 2018). These regulatory factors within the MSC-derived exosomes are reported to act on critical cellular pathophysiological processes, such as energy metabolism, inflammation, and migration (Nakano et al., 2016; Borger et al., 2017). Li et al. (2017) reported that MSC-oriented exosomes shift the M1 microglia polarization toward an M2 phenotype, which ameliorates neuroinflammation and promotes functional recovery in a TBI model. Nakano et al. (2016) reported that BM-MSC-derived exosomes can alleviate the learning and cognitive damage in Streptozotocin (STZ)-induced diabetic mice by alleviating oxidative stress and promoting synaptogenesis. Functional miRNAs in exosomes might be an important mediator to inhibit neuronal apoptosis, promote synaptic plasticity and remodeling, and accelerate functional recovery (Xin et al., 2013, 2017; Cheng et al., 2018). For example, Xin et al. (2012, 2013) reported that MSC-released exosomes can transfer miR-133b into astrocytes and neurons in stroke rat models, and thereby promote the neurite outgrowth in the ischemic area. The authors further demonstrated the miR-133b exerts their neuroprotective function *via* decreasing the Ras homolog gene family member A (RhoA) and connective tissue growth factor (CTGF) expression. Besides, a study based on miRNA profiling and qPCR demonstrated the upregulation of miR-146a-5p in senescent MSCs and MSC-MVs (Lei et al., 2017). MiR-146a were also significantly upregulated in peripheral blood and cerebral spinal fluid (CSF) during the progression of AD, amyotrophic lateral sclerosis (ALS), and MS (Viswambharan et al., 2017). The miR-146a transferred by BM-MSCs-secreted exosomes is a key regulator in suppressing the astrocytic inflammation and protecting diabetic rats from cognitive decline (Kubota et al., 2018). Other neuroprotective mechanisms of MiR-146a-5p include inhibiting the NF-κB signaling (Iyer et al., 2012) and promoting remyelination (Zhang et al., 2017).

However, the neuroprotective functions of MSCs may be restricted with age, since both the number and function of MSCs are decreased (Lee et al., 2010; Bang et al., 2016). The senescent MSCs commonly present enlarged and more granular morphology, limited proliferation and differentiation capacity, and a distinct secretory phenotype referred to as “senescence-associated secretory phenotype” (SASP; Watanabe et al., 2017). The senescence of MSCs is believed to be the underlying cause of osteoporosis (Román et al., 2017), and neurodegenerative diseases including AD and PD (Lei et al., 2017). Furthermore, MSCs in the senescent late period release higher levels but smaller-sized MSC-MVs than the early passage of MSCs (Lei et al., 2017), which correlated with the phenomenon that the levels of myeloid MVs in plasma and cerebrospinal fluid were positively associated with neurodegenerative disease (Joshi et al., 2014).

In conclusion, BM-MSCs can migrate into the brain to differentiate toward neurons themselves, or recruit microglia cells for toxicant clearance. BM-MSCs can also promote neuronal repair and functional recovery by secreting exosomes. However, BM-MSC’s curative effects decreased greatly in the aging process.

## BM-Controlled Immune System

**Figure 2:**
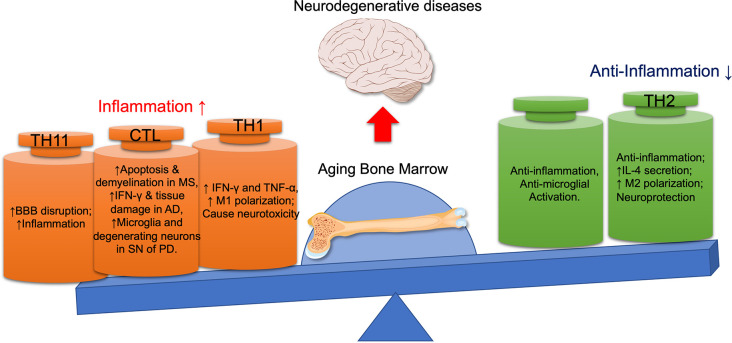
The detrimental and beneficial roles of different immune cells in neurodegenerative diseases. With the aging bone marrow, pro-inflammatory lymphocytes (CTL, Th1, Th17) are activated, while immunomodulatory lymphocytes (Th2, Tregs) are suppressed, thus promoting the progression of neurodegenerative diseases. AD, Alzheimer’s disease; BBB, blood-brain barrier; CTL, cytotoxic T lymphocyte; IFN-γ, interferon-γ; MS, multiple sclerosis; PD, Parkinson disease; SN, substantia nigra; TNF-α, tumor necrosis factor-α.

The BM is the primary organ for producing common lymphoid progenitors (CLPs), which are responsible for generating innate and adaptive immune cells (Zhao E. et al., 2012). This is why the BM is called the primary lymphoid organ. The function of the BM and CNS is closely interconnected. There are norepinephrine (NE)-releasing sympathetic nerve fibers in BM, which are essential for preserving the HSC niche, promoting the HSC mobility, and regulating their differentiation into immune cells (Maryanovich et al., 2018). At the same time, the changes in the immune system originated from the BM will also affect the functions of the CNS by various mechanisms, including promoting inflammatory cytokines secretion, microglial activation, neuronal apoptosis, demyelination, and tissue damage (Liang et al., 2017). With age, the hematopoietic tissue is gradually replaced by fat tissue, and the proliferative and developmental capacity of HSCs, as well as the lymphogenic capacity decrease (Geiger et al., 2013). Studies have shown that the pathogenesis of AD and PD may be related to systemic immune dysfunction, especially to the T cell subtypes (Pellicano et al., 2012). Compared with the age-matched controls, the difference of immunity in AD patients was greater than that caused by age. The immune alterations mainly include less naïve T cells, more terminated-differentiated cells, and more dysfunctional Treg cells in AD patients. In PD patients, CD4+ and CD8+ T lymphocytes are found aggregated around the SN compacta (Brochard et al., 2009). These CD4+ cells are highly pro-inflammatory, which can promote the microglia activation and the degeneration of dopaminergic neurons. These studies suggest that immune alterations can contribute to the progression of neurodegenerative diseases (Figure 2).

### CD8^+^ T Cells

CD8+ T cells also referred to as cytotoxic T lymphocytes (CTL), play vital roles in the immune system to defend against intracellular pathogens by secreting pro-inflammatory cytokines, primarily the TNF-α and IFN-γ, or directly killing target cells *via* Fas/FasL interactions or cytotoxic granules containing perforin and granzymes (Harty et al., 2000). CD8+ T cells are also related to the pathogenesis of neurodegenerative diseases. Studies indicated that the CD8+ T cells outnumber the CD4+ T cells in MS lesions (Goverman et al., 2005). Moreover, the injection of CD8+ myelin-specific T cells rather than myelin-specific CD4+ T cells into wild-type mice can induce a demyelinating disease similar to MS, suggesting CD8+ T cells’ unique roles in the pathogenesis of MS (Huseby et al., 2001; Goverman, 2009). Furthermore, in the brains of both postmortem human PD specimens and PD mouse models, CD8+ T cells are often located next to the activated microglia and degenerating neurons in the SN area, which indicates that these cells might be implicated in PD neuronal loss (Brochard et al., 2009). Additionally, CD8+ T cells mediated inflammation and IFN-γ levels are associated with microstructural tissue damage and neurological deficits in AD patients (Baglio et al., 2013; Lueg et al., 2015).

### Th1 Cells

CD4+ Th1 cells referred to as type 1 T helper cells are characterized by T-bet expression and Type 1 cytokines (mainly IFN-γ, TNF-α, and IL-2) secretion (Romagnani, 2014). CD4+ Th1 infiltration in the brain is seen in various neurodegenerative diseases (Liang et al., 2017). Browne et al. (2013) showed that Th cells infiltrated in the brains of APP/PS1 mouse models secrete abundant IFN-γ and IL-17, suggesting these Th cells are mainly the Th1 and Th17 subsets. They further demonstrated that it is the Th1 cells, but not Th2 nor Th17 cells, that cause microglial activation, Aβ plaque burden increase, and impaired cognitive function in APP/PS1 mouse models (Browne et al., 2013). Much of the Th1 cell-mediated inflammatory damage in the context of neurodegenerative diseases can be attributed to the release of pro-inflammatory cytokines and the M1 polarization of macrophages and microglia. These cellular processes could significantly aggravate neurodegenerative diseases by promoting neurotoxicity and tissue damage in the brain (Sanchez-Guajardo et al., 2013). Administrating anti-IFN-γ antibody and immuno-modulative TGF-β in AD mouse models could effectively alleviate the Th1 cell-mediated neuroinflammation and cognitive decline (Browne et al., 2013; Chen et al., 2015). However, some studies show the beneficial roles of Th1 cells in AD pathology. Intracerebroventricular (ICV) injection of Aβ-specific Th1 cells lowers the Aβ levels and enhances neurogenesis, while exerts no impact on apoptosis in AD mouse models (Fisher et al., 2014). Thus, it seems that different routes of Th cells migration into the brain might play different roles in neurodegeneration.

### Th2 Cells

CD4+ Th2 cells are T helper cells expressing the GATA3 and producing Type-2 cytokines (mainly IL-4, IL-5, IL-9, and IL-13; Walker and McKenzie, 2018). Th2 cells mainly exhibit an anti-inflammatory function, such as suppressing the Th1-related IFN-γ-driven immune response and inducing macrophage to polarize into an M2-like phenotype (Walker and McKenzie, 2018). In the Th1/Th2 balance paradigm, the Th1-secreted IFN-γ and Th2-derived IL-10 inhibit the proliferation of each other’s cells. CD4+ Th2 cells mainly exhibit neuroprotective properties in the context of neurodegenerative diseases. For example, myelin basic protein (MBP)-primed Th2 cells can enter the brain and restrict the AD and PD neurotoxicant induced microglial inflammation (Roy and Pahan, 2013). In MS models, Th2 cells can inhibit the activation of lipopolysaccharide (LPS)-stimulated microglia *via* direct cell contact, and ultimately inhibit its IL-1b and nitric oxide production (Roy and Pahan, 2013). Additionally, MBP-primed Th2 cells can even induce the neurotrophic molecules (including BDNF and NT-3) expression in microglia and astroglia *via* direct cell contact (Roy et al., 2007).

### Th17 Cells

Th17 cells are CD4+ Th cells expressing the RORγτ transcriptional factors and secreting characteristic cytokines including IL-17 and IL-22 (Annunziato et al., 2013). They are involved in the progression of neurodegenerative diseases, mainly by secreting IL-17 to recruit inflammatory neutrophils and IL-22 to stimulate epithelial cells generating antimicrobial peptides (Saresella et al., 2011). Saresella et al. (2011) analyzed the function of various T lymphocytes in AD patients and compared the data with those of mild cognitive impairment participants or aged-matched healthy people. They found that the Th17 lymphocytes are significantly increased in AD. In MS or experimental autoimmune encephalomyelitis (EAE), Th17 cells secrete cytokines to act on IL-17 and IL-22 receptors on the BBB endothelium, causing BBB damage and subsequent neural inflammation (Kebir et al., 2007).

Interestingly, Th17 is tightly associated with bone destruction. In postmenopausal osteoporosis patients, serum IL-17A is significantly higher, while the IFN-γ and IL-4 are significantly lower, suggesting osteoporosis may be more associated with the Th17 cells rather than the Th1 or Th2 cells (Zhang et al., 2015). The further study demonstrates IL-17A is pro-osteoclastogenic at the cellular level (Le Goff et al., 2019). *In vivo* study shows that although IL-17A is not required for normal bone homeostasis, it plays a vital role in bone loss of ovariectomized osteoporosis mouse models. Furthermore, preliminary data from clinical trials show that anti-IL-17A antibodies stabilize bone density in inflammatory arthritis (Le Goff et al., 2019). These results suggest an inter-relationship between osteoporosis and neurodegenerative diseases.

### Treg Cells

Regulatory T cells (Tregs) are T cells expressing the CD4+CD25+Foxp3+ surface markers. They inhibit immune response and thus to maintain homeostasis and self-tolerance. Treg cells play a substantial role in slowing down the progression of ALS by lowering pro-inflammatory cytokine expressions and reducing microglial activation in mouse models (Beers et al., 2011). The mechanisms behind are associated with Tregs’ secretion of immuno-modulative cytokines (TGF-β, IL-10, and IL-4) which inhibits the microglia and pro-inflammatory T cells activation (Beers et al., 2011; Xie et al., 2015). However, functional Tregs are usually deficient in patients with neurodegenerative diseases for several reasons. Under normal physiological conditions, 30% of CD4^+^ T cells are functional Treg cells in BM (Zou et al., 2004), and over 15% of CD4+ T cells in rat cerebrum are Treg cells (Xie et al., 2015). However, the absolute lymphocyte numbers and the percentage of Treg subsets were both decreased in the BM and peripheral blood of the aged participants, indicating the Treg production in BM is reduced (Freitas et al., 2019). Moreover, a decrease of functional Treg seems to be closely related to MS, since the relapsed MS patients have fewer Tregs and significantly reduced Treg/Th17 ratio in the peripheral blood compared with healthy individuals (Jamshidian et al., 2013). Furthermore, Tregs are extremely rare or even undetectable in MS brain lesions, which might be due to Treg’s impaired migration across the BBB (Fritzsching et al., 2011). *In vitro* studies show healthy Tregs can cross the human brain endothelium easily, whereas the migration ability of Tregs in MS patients is severely impaired (Schneider-Hohendorf et al., 2010). Moreover, their neural protective functionality might also be hindered in neurodegenerative disease because of the Th17 subset’s inhibitory effect on Tregs. Treg cells rely on TGF-β, which is competitively bound by the abundant Th17 cells in a neurodegenerative context, for differentiation and maintenance (Afzali et al., 2010). However, Tregs can be transformed into pro-inflammatory Th17 cells themselves in some circumstances (Afzali et al., 2010). These multiple factors result in low Treg levels in neurodegenerative patients.

## Bone Secretory Proteins

Previous evidence showed that several bone cell-secreted hormones or “osteokines” have endocrine functions, such as OCN, OPN, FGF23, Lipocalin 2(LCN2), osteoprotegerin (OPG), sclerostin (SOST), and Dickkopf-1 (DKK1; Vervloet et al., 2014; Han Y. et al., 2018). Most of these proteins are produced by osteoblasts and osteocytes and have roles in regulating phosphate and systemic energy metabolism (Han Y. et al., 2018). Osteoblasts-secreted OCN regulates energy metabolism, reproduction, and cognition (Zoch et al., 2016; Mizokami et al., 2017; Obri et al., 2018). OPN in the osseous tissue is released from osteoblasts and osteoclasts and is associated with bone destruction and suppression of ectopic calcification (Lund et al., 2009; Uede, 2011). It mainly regulates phosphate homeostasis (Huang et al., 2013). LCN2 secreted by osteoblasts can act on the brain to suppress appetite (Mosialou et al., 2017). OPG is synthesized by osteoblasts and its main function is to antagonize the effects of receptor activator of nuclear factor-kappa-B ligand (RANKL; Bonnet, 2017; Rochette et al., 2019). Both SOST and DKK1 (Ke et al., 2012) are mainly secreted by osteocytes and function through antagonizing the canonical Wnt pathway. These bone secretory proteins are reported to play diverse roles in neurodegenerative diseases (Table 1).

**Table 1:** Bone secretory proteins and their different effects in the brain.

	Origin	Neuroprotective effects on the brain	Neurotoxic effects on the brain
OCN	Osteoblast	↑MN (5-HT, DA, NE) and ↓ GABA production; ↑BDNF; ↓Inflammation; Beneficial for AD and PD.	
OPN	Osteoblasts, osteoclasts, myelomonocytic cells	↑Monocyte-macrophage’ recruitment and M2 polarization in AD; ↑Aβ clearance in AD; ↓Apoptosis in AD; Either neuroprotective or neurotoxic in PD; Protects dopaminergic cells in PD *via* RGD-binding domain.	↑Pro-inflammatory mediators in MS; ↑Autoreactive immune cells in MS; Associate with nigral cell death and glial response in PD.
FGF23	Osteoblasts, osteocytes		↓Vitamin D, exacerbate AD and PD; ↓Serum phosphate, cause ion dysregulation; FGF23 deficiency causes ectopic calcification, neuronal loss, and cognition damages.
LCN2	Osteoblast		Inhibit remyelination in MS; Synergistic neurotoxic effects with Aβ, TNF-α or LPS; ↑Apoptosis and neuronal death in MS, PD, AD; ↓TNFR2’s neuroprotective effects.
OPG	Osteoblast	Inhibit RNAKL and TRAIL; ↓Aβ toxicity and prevent AD; ↓Vascular calcification and prevent vascular dementia.	
DKK1	Osteocytes, osteoblasts, BM-MSCs		↓Wnt’s neuroprotective effects; ↑Aβ toxicity; ↓Neural synapses; Risk factor for AD.

*Abbreviations: OCN, osteocalcin; OPN, osteopontin; OPG, osteoprotegerin; FGF23, fibroblast growth factor 23; LCN2, Lipocalin 2; DKK1, Dickkopf-1; MN, Monoamides; 5-HT, serotonin; DA, dopamine; NE, norepinephrine; GABA, γ-aminobutyric acid; RGD, arginine-glycine-aspartic acid; RANKL, receptor activator of the nuclear factor-kappa-B ligand; TRAIL, TNF-related apoptosis-inducing ligand; BDNF, brain-derived neurotrophic factor; AD, Alzheimer’s disease; MS, multiple sclerosis PD, Parkinson’s disease; TNFR2, tumor necrosis factor receptor 2; LPS, lipopolysaccharide; TNF-α, tumor necrosis factor-α*.

### OCN

OCN is uniquely secreted by osteoblasts (Hauschka et al., 1975; Price et al., 1976) and is thus a specific biomarker for bone formation (Ducy et al., 1996; Ducy, 2011). OCN is also an important regulator of energy metabolism, as it can enhance insulin secretion, decrease insulin resistance, improve glucose tolerance and blood lipid profile, and regulate brown adipose tissue differentiation (Karsenty and Ferron, 2012; Wei et al., 2014).

OCN, uncarboxylated in most circumstances, can pass through the BBB and bind specifically with neurons in the brainstem, thalamus, and hypothalamus (Oury et al., 2013; Shan et al., 2019). Upon binding, OCN can influence the signals that regulate neurotransmitter syntheses, such as decreasing the synthesis of glutamate decarboxylase 1 (Gad1), an enzyme involved in GABA biosynthesis, and increasing the tyrosine hydroxylase (Th) and tryptophan hydroxylase 2 (Tph2) synthesis, which are the key enzymes involved in the serotonin, dopamine, and norepinephrine generation in the brainstem and midbrain explants (Oury et al., 2013). In the hippocampus, OCN mainly combines with neurons’ Gpr158/Gaq receptors, and functions in part by activating IP3 and promoting the secretion of BDNF (Khrimian et al., 2017), a molecule well known to promote hippocampal-dependent memory (Hall et al., 2000; Dean et al., 2009). Adult mice lacking OCN displayed a substantial increase in anxiety-like behavior and had a major deficit in memory and learning (Nakazawa et al., 2002; Oury et al., 2013). Anatomically, the brains of OCN–/– mice are consistently smaller, mainly in the hippocampal region, and often lost the corpus callosum compared with wild-type littermates (Nakazawa et al., 2002; Oury et al., 2013). OCN–/– mice injected with OCN showed ameliorated anxiety and depression, and improved memory and learning abilities (Mera et al., 2016). OCN also has neuroprotective effects in the context of PD. As mentioned above, OCN could enhance dopamine synthesis in neurons, thereby reducing the Th loss and relieving the PD symptoms in PD rat models (Guo et al., 2018; Obri et al., 2018). Also, OCN could modulate neuroinflammation in the SN of PD rats by inhibiting astrocyte and microglia proliferation, together with partially decreased levels of TNF-α and IL-1β (Guo et al., 2018).

### OPN

OPN was first discovered as a protein to anchor osteoclast to the mineral surface of bones, thereby to facilitate the osteolytic process (Reinholt et al., 1990). Serum OPN levels have negative correlations with bone mineral density in postmenopausal women (Cho et al., 2013). OPN is mainly secreted by osteoblasts, osteoclasts, and BM-derived myelomonocytic cells (Lund et al., 2009; Uede, 2011). In the brain, OPN is a constituent of the normal extracellular matrix and is expressed mainly in the basal ganglia, especially in the substantia nigra (SN; Iczkiewicz et al., 2004). In neurodegenerative diseases, OPN is considered to play dual roles in neuroinflammation and neuroprotection (Carecchio and Comi, 2011; Yu et al., 2017).

Previous studies showed OPN’s detrimental role in MS. Abundant OPN transcript was found in plaques dissected from MS patients’ brains, while it was absent in the control group (Chabas et al., 2001). Further study demonstrated that OPN-deficient mice had a milder disease course than wild type animals, and displayed only a single relapse without subsequent exacerbations or progression (Chabas et al., 2001; Jansson et al., 2002). Administrating OPN-deficient EAE mice with recombinant OPN exacerbates the disease (Hur et al., 2007). Two mechanisms may be involved in OPN’s detrimental effects: by stimulating the pro-inflammatory mediators in MS lesions, and by inhibiting the apoptosis of autoreactive immune cells (Carecchio and Comi, 2011). In contrast, the OPN is mainly studied for its anti-inflammatory and anti-apoptotic properties in PD (Khan et al., 2002; Lund et al., 2009; Rittling and Singh, 2015). Iczkiewicz et al. (2010) showed that the arginine-glycine-aspartic acid (RGD)-binding domain of OPN protects dopaminergic cells against toxic insult induced by MPP+ and LPS. Also, the OPN’s effect decline with age, a major predisposing factor for PD, further reinforced the hypothesis (Hwang et al., 1994). However, Maetzler et al. (2007) showed that OPN knock-out PD mice displayed less nigral cell death and a lower glial response compared to wild-type PD mice. He also reported that PD patients’ serum and CSF OPN levels were higher, with CSF levels positively correlated with concomitant dementia and serum levels with more severe motor symptoms, suggesting OPN may promote the PD progression (Maetzler et al., 2007). In the context of AD, studies show OPN can promote the monocyte-macrophage’ recruitment into AD mouse brains, and their polarization towards an anti-inflammatory, highly phagocytic phenotype to facilitate Aβ clearance (Rentsendorj et al., 2018). The FDA-approved anti-AD drug glatiramer acetate increases the plasma levels of OPN, and therefore promotes a macrophage phenotype that is highly phagocytic of Aβ and anti-inflammatory (Rentsendorj et al., 2018). Also, OPN can bind to the CD44 receptor and exert its anti-apoptotic function (Lin and Yang-Yen, 2001). This may be another potential mechanism for OPN to protect neurons from injury in AD.

Therefore, we can conclude that the levels of OPN increase with age. OPN accelerates the progression of bone demineralization, while exerts different influence on various kinds of neurodegenerative disorders.

### FGF-23

FGF-23 is mainly secreted by osteoblasts and osteocytes. It suppresses the phosphate resorption and 1, 25(OH)2D3 production in the kidney by binding to FGFR1 and its co-receptor Klotho (Urakawa et al., 2006). FGF-23 changes significantly in the aging process (Cardoso et al., 2018). Still, FGF-23 is an independent predictor for dementia and AD, after adjusting for age, sex, cardiovascular disease, diabetes mellitus, etc. (McGrath et al., 2019). In another study, researchers used high-resolution MRI to find out that increased FGF-23 was associated with axonal loss and white matter disruption in the frontal lobe only in patients with cardiovascular risk factors (Marebwa et al., 2018). FGF-23-deficient mice show ectopic calcifications in the brain, fewer immature neurons in the sub-granular zone (SGZ), and significant cognitive impairment compared with wild type controls (Kunert et al., 2017; Laszczyk et al., 2019). Mice overexpressing FGF-23 also showed impaired spatial learning and memory (Liu et al., 2011). However, these peripheral symptoms could be largely alleviated by dietary correction of phosphate levels (Morishita et al., 2001; Liu et al., 2011), suggesting that FGF-23 may indirectly affect brain health and cognition, but probably by affecting phosphate homeostasis which acts synergistically with renal or cardiovascular risk factors.

Another explanation is that too much FGF-23 may exacerbate neurodegenerative diseases by decreasing the level of vitamin D3. FGF-23 suppresses the vitamin D3 production by blocking the 1α-hydroxylase in the renal proximal and distal tubules (Karsenty and Olson, 2016). The detrimental effects of vitamin D deficiency on the brain and its roles in neurodegenerative diseases have already been elaborated on in other studies (Koduah et al., 2017; Lv et al., 2020).

In conclusion, an abnormal amount (too much or too little) of FGF-23 is sufficient to affect brain function and cognition, most likely through an indirect way by affecting ion (phosphate) and/or vitamin D homeostasis.

### LCN2

LCN2 is an endocrine hormone secreted by osteoblasts and can suppress appetite by crossing the BBB to bind to the melanocortin-4 receptor (MC4R) in the brainstem (Mosialou et al., 2017). Besides, LCN2 can promote insulin resistance and cause hyperglycemia, diabetes, cardiovascular disease, and metabolic syndrome (Yan et al., 2007). LCN2 can also be secreted by neutrophils and glial cells, and play essential roles in inflammation, infection, and injury to cells (Pinyopornpanish et al., 2019).

LCN2 acts through its two receptors, 24p3R and megalin (Chakraborty et al., 2012), both of which can be found in the brain in basal and pathological conditions (Ip et al., 2011). LCN2 production is increased in progressive MS patients, and this effect could be relieved by the MS-treating drug natalizumab (Al Nimer et al., 2016). Further *in vitro* study shows that LCN2 plays detrimental roles by inhibiting remyelination in a dose-dependent manner (Al Nimer et al., 2016). LCN2 is also upregulated in the SN of PD patients and neurotoxin-induced PD animal models (Kim et al., 2016). The higher LCN2 levels could disrupt the SN dopaminergic projection and contribute to abnormal locomotor behaviors through neurotoxic iron accumulation and neuroinflammation, which were alleviated in LCN2-deficient mice (Kim et al., 2016). LCN2 is significantly decreased in CSF of AD patients and increased in brain regions related to AD pathology (Naudé et al., 2012). *in vitro* study demonstrates that LCN2 makes nerve cells susceptible to Aβ toxicity, and suppresses the neural protective tumor necrosis factor receptor 2 (TNFR2) signaling pathway in neurons (Naudé et al., 2012).

In animal experiments, ICV injection of recombinant mouse LCN2 protein can cause neuronal death in the hippocampal CA1 area and cognitive dysfunction (Kim et al., 2017). Moreover, LCN2 usually acts synergistically and exacerbates the neurotoxic effects of Aβ, TNF-α, or LPS (Mesquita et al., 2014; Yang et al., 2017). The expression of 24p3R increases after the administration of Aβ (Mesquita et al., 2014). An increase in cell death is reported when astrocytes or neurons are co-cultured with LCN2 and Aβ (Marebwa et al., 2018), and the depletion of LCN2 helps to protect astrocytes from Aβ toxicity (Mesquita et al., 2014). Also, LCN2 could undermine the neuroprotective effect of the TNFR2 signaling pathway induced by TNF-α (Hemmings and Restuccia, 2012; Naudé et al., 2012). Further *in vitro* studies demonstrate that LCN2 can directly induce neuronal apoptosis through the BCL2 mediated cell death signaling pathway in a time and dose-dependent manner (Lee S. et al., 2012; Bi et al., 2013).

In short, LCN2 could exert pro-apoptotic effects on brain cells and increase the susceptibility of neurons to toxic stimuli. LCN2 could also increase glial activity, increase inflammation, and inhibit remyelination.

### OPG

OPG is a soluble glycoprotein that belongs to the TNF receptor superfamily. It is a decoy receptor for RANKL and TNF-related apoptosis-inducing ligand (TRAIL), and thus inhibits the association of RANKL and TRAIL with their receptors. It is secreted by osteoblast and prevents the RANKL from binding to its receptor on osteoclasts, thereby inhibiting the osteolysis (Bonnet, 2017; Rochette et al., 2019). Increased blood levels of OPG are associated with osteoporosis in postmenopausal women (Yano et al., 1999).

High OPG levels are also detected in the CSF, and as the OPG level in the CSF increases with age, it may play a role in inflammatory and degenerative disorders of the CNS (Hofbauer et al., 2004). A study demonstrates that after adjusting for age, sex, and APOE ε4 allele, OPG is an independent predictor of AD and vascular dementia (Emanuele et al., 2004). OPG might influence cognition by affecting the immune environment and the perfusion of the brain. For the former, studies have demonstrated that the RANKL/RANK signaling suppresses inflammation through a Toll-like receptor pathway in microglia. Increased OPG could inhibit the RANKL/RANK signaling and aggravate the post-ischemic inflammation (Shimamura et al., 2014). Whereas for the latter, the RANK/RANKL/OPG triad might play a critical role in vascular calcification (Rochette et al., 2019). Therefore, OPG can prevent vascular calcification as a RANKL inhibitor (Wu et al., 2013). Emanuele et al. (2004) measured plasma OPG levels in vascular dementia patients and compared them with OPG in AD and age-matched healthy individuals. They found that compared with the non-demented control group, OPG concentrations were significantly higher in both vascular dementia and AD patients, wherein the OPG level in vascular dementia was the highest. These results were interpreted to be that OPG could mirror atherosclerotic disease, most so in vascular dementia and that vascular factors may also play a role in the pathogenesis of AD (Emanuele et al., 2004). Increases of OPG in vascular dementia can be regarded as a compensatory defense mechanism to relieve the atherosclerotic burden (Schoppet et al., 2002).

### DKK1

Both DKK1 and SOST are soluble Wnt inhibitors (Ke et al., 2012). Although these two molecules share some homologies in action, they have distinct biological effects and different expression patterns (Gifre et al., 2013). The DKK1 and SOST use a different receptor to inhibit the low-density lipoprotein receptor-related protein (LRP)5-LRP6 receptor complex formation (Monroe et al., 2012). The SOST protein seems to be a specific Wnt inhibitor because it is almost exclusively bone-derived, and is predominantly secreted by osteocytes and osteoclast precursors (Poole et al., 2005; Vervloet et al., 2014). Although DKK1 is also mainly expressed by osteocytes, osteoblasts, and BM-MSCs, it is not as highly selective as SOST (Han Y. et al., 2018). We mainly focus on the DKK1 in this review, since it is more deeply involved in brain pathologies, while the SOST is found unrelated to cognition change (Ross et al., 2018).

Wnt signaling has been proven to have strong neuroprotective effects, and can even regulate neo-neuronal generation in the adult brain (Inestrosa and Varela-Nallar, 2014). Wnt signaling activation facilitates synaptic remodeling and memory consolidation (Inestrosa and Varela-Nallar, 2014). Therefore, it is easy to understand that Dkk1, a Wnt signaling inhibitor, is associated with the severity of neurodegenerative diseases (Ross et al., 2018). The expression of DKK1 in the brain tissue, CSF, and plasma of AD patients and animal models increases significantly compared to healthy controls (Caricasole et al., 2004; Rosi et al., 2010). It has also been reported that at high concentrations, DKK1 can pass through the BBB, and thus accelerate the AD progression (Ren et al., 2019). Some studies show that the occurrence of familial/early-onset AD, sporadic/late-onset AD, and patient’s cognitive decline are related to DKK1 and Wnt/β-catenin signaling disruptions (Scott and Brann, 2013). The increased expression of DKK1 inhibits the Wnt signaling pathway, which further increases the tau phosphorylation, while the DKK1 knock-out inhibits the formation of neurofibrillary tangles, and reduces the neurotoxicity of Aβ (Caricasole et al., 2004). Further study shows that injecting different concentrations of DKK1 into the dorsal hippocampus can lead to object recognition memory loss, which is regulated by the canonical Wnt-dependent signaling pathway (Fortress and Frick, 2016). DKK1 could also aggravate the Aβ-induced neuronal apoptosis and synaptic loss by blocking the Wnt signaling pathway (Purro et al., 2012). Other related studies show that short-term exposure to Aβ in mouse brain slices increases the DKK1 expression and reduces the synaptic formation (Matrisciano et al., 2011), while injecting DKK1-specific antibodies could alleviate the Aβ-induced damage to neuronal synapses (Huang et al., 2018). Some scholars suggest that the endogenous Wnt ligands, which are inhibited by DKK1, are vital to maintaining the neuronal synapses (Purro et al., 2014). Also, the potential therapeutic effects of the DKK1 inhibitor may be associated with synaptic preservation and hippocampal circuit reconstruction (Marzo et al., 2016; Ortiz-Matamoros and Arias, 2018). Animal tests show that inhibiting DKK1 improves subjects’ spatial memory tasks (Marzo et al., 2016; Ortiz-Matamoros and Arias, 2018) and their electrophysiological findings (Marzo et al., 2016).

## Potential Therapeutic Implications

### BM-Derived Cells

After the transplantation of BM-MSCs, learning ability and spatial memory performance were significantly improved in AD animal models (Ohsawa et al., 2008; Kanamaru et al., 2015; Safar et al., 2016). It was revealed that the gene expression patterns are altered in AD brains, and the alterations are correlated with the severity of neuropathology (Qin et al., 2020). Transplantation of BM-MSCs could also alter some gene levels in the AD brains, such as genes related to pro-inflammatory cytokines, enzymes, receptors, and intermediate filaments. These differentially expressed representative genes are mostly responsible for neuropathological phenotypes in AD (Qin et al., 2020).

In terms of feasibility, the intravenous delivery of stem cells is convenient and sufficient to lower the levels of cerebral amyloidosis (Salem et al., 2014; Harach et al., 2017). The intravenous transplanted stem cells could be easily detected in the brain parenchyma, i.e., could be found in the hippocampus 1 h after administration (Harach et al., 2017), and expressed neuronal phenotypes in the brain for 1-6 months (Brazelton et al., 2000). The expression of male BM-MSCs’ *sry* gene in female AD model’s brain tissue proves that exogenous stem cells with intravenous delivery could successfully migrate to the brain injury site (Salem et al., 2014). As for the cell dose for intravenous administration, our previous data showed 5 × 10^5^ BM-MSCs is an appropriate dose for treating ischemic stroke in rats (He et al., 2016). The optimal cell dose for AD and other neurodegenerative disease therapies are remained to be explored.

### Bone-Derived Exosomes

Previous studies report positive correlations between MVs in CSF and neurodegenerative diseases and show that many exosomes are enriched with undigested lysosomal substrates, such as Aβ and APP (Malm et al., 2016). However, these MVs are mainly derived from inflammatory cells, microglia, or tumor (Rajendran et al., 2006; Saman et al., 2012, 2014; Joshi et al., 2014; Levy, 2017), and there is no evidence suggested that BM-derived exosomes can cause or exacerbate neurodegenerative diseases. Some studies indicate that using stem cell-secreted exosomes as an alternative therapeutic would be much safer compared with stem cell transplantation (Smith et al., 2020). Administrating exosomes rather than viable replicating cells can mitigate many complications and therefore be much safer (Smith et al., 2020). The transplanted stem cells may persist or be amplified even after treatment is terminated. The transplanted stem cells’ potential of differentiating into other cell types has also raised long-term safety concerns (Breitbach et al., 2007).

Apart from its innate therapeutic effect discussed above, exosomes from the BM can be modified into appropriate nanocarriers to transport siRNA. To strengthen exosomes’ targeting ability, researchers reshaped them by attaching neuron-specific Rabies Viral Glycoprotein (RVG) peptides onto their surface (Alvarez-Erviti et al., 2011). The remodeled RVG exosomes successfully transported the GAPDH siRNA into targeted neurons, oligodendrocytes, and microglia, and subsequently downregulated the BACE1 gene expression, which is crucial in the AD progression.

### Bone Secretory Proteins

Several bone-secreted proteins were summarized above as regulators of CNS homeostasis and neurodegenerative diseases. Although the direct use of these bone-secreted proteins as drugs to treat neurodegenerative diseases are rare, a few studies indicated that these proteins might be indirectly involved in other therapeutics. For example, metformin could ameliorate spatial memory loss (Ahmed et al., 2017), reduce stress-induced behaviors (Mourya et al., 2018), and relieve the related symptoms in the PD mouse model (Katila et al., 2017). Metformin can affect OCN and related bone diseases. In an osteoporosis mouse model, metformin could ameliorate the bone loss with the increased OCN expression level (Liu et al., 2019). The drug could also improve the osteogenic functions of adipose-derived stromal cells by increasing the OCN expression and AMPK signaling (Smieszek et al., 2018). It should be noted that AMPK is also a regulator for brain energy metabolism (Garza-Lombó et al., 2018), which are related to cognition and motor coordination (Kobilo et al., 2014), and can inhibit NF-κB signaling and inflammation (Salminen et al., 2011). Moreover, metformin can behavior by increasing the BDNF secretion (Katila et al., 2017; Fatemi et al., 2019), which coordinates with OCN’s effect in age-related memory loss (Khrimian et al., 2017). These findings suggested that metformin therapy could influence brain functions, wherein the OCN may act as a mediator in promoting neurotrophic signal, energy metabolism, and immune modulation. A role for OCN may also be implicated in exercise-induced cognitive improvement through the IL-6/gp130/OCN axis (Shan et al., 2019). Moreover, the MS-treating drug natalizumab and the anti-AD drug glatiramer acetate mentioned above are another two examples to treat neurodegenerative diseases *via* the mechanisms related to bone secretory proteins. The two drugs reduce the LCN2 or increase the OPN expression, respectively.

Before clinical application, further studies on specific bone regulative processes and their effects on the brain are required. For example, even though BM-derived cells can enter the CNS and become neural progenitors or microglia-like cells, more details about how this process is controlled *in vivo* should be elucidated (Ajami et al., 2007; Mildner et al., 2007). Moreover, most studies examining the bone-derived cells or molecules’ roles in neurodegenerative diseases were performed in mouse models rather than in patients. These results should further be confirmed in human samples. Furthermore, except for OCN and DKK1, there is still a lack of direct evidence to show that these bone-secreted osteokines can cross the BBB and influence the neurodegenerative diseases’ course. Although these cytokines are mainly secreted from the bone, and show altered expression in the context of neurodegenerative diseases, most osteokines above are not bone-specific and some of them can be secreted within the brain. It remains to be determined how these osteokines are evolved in the process of bone-brain crosstalk and contribute to the progression of neurodegenerative diseases by using bone-defect models. Last but not least, although bone is a primary lymphoid organ, it does not sufficiently control the entire immune system, other factors are also needed for triggering the onset of neurodegenerative diseases. Although there are still many difficulties, the potential application of bone-based therapy in neurodegenerative diseases is worthy of further experimental and clinical studies.

## Conclusions

Elderly people are prone to osteoporosis and neurodegenerative diseases including cognitive decline, PD, and MS. All these disorders have common soil, mainly the metabolic-related disorders and immune dysregulation, suggesting the bone and brain may be closely interacted. When anyone of the two sides breaks, the whole interconnected balanced system collapses. Finding out the interactive mechanisms and looking for early detection and intervention may be a promising way to prevent the disease progress. This review summarizes the roles of the bone-brain axis in the progression of neurodegenerative diseases. We focus on the effects of BM-derived cells, mainly the microglia-like cells and MSCs, the BM-controlled immune system, and the bone secreted proteins on the brain. Evidence from experimental studies is encouraging, wherein bone-derived cells and factors can influence brain development, neurotransmitter synthesis, ion homeostasis, neuroinflammation, and neurotoxicant clearance. Therefore, targeting and modulating bone physiological processes can be promising for developing novel therapeutic approaches for neurodegenerative diseases.

## Author Contributions

ZY drafted the article. ZL and LL revised it critically for important intellectual content. JZ and XC contributed to the revision of the manuscript and figure illustrations. XZ and PX made substantial contributions to the conception and design of the review and gave final approval of the version to be published. All authors contributed to the article and approved the submitted version.

## Conflict of Interest

The authors declare that the research was conducted in the absence of any commercial or financial relationships that could be construed as a potential conflict of interest.
